# Electric Field Effects on Brain Activity: Implications for Epilepsy and Burst Suppression

**DOI:** 10.3390/cells12182229

**Published:** 2023-09-07

**Authors:** Evan D. Doubovikov, Natalya A. Serdyukova, Steven B. Greenberg, David A. Gascoigne, Mohammed M. Minhaj, Daniil P. Aksenov

**Affiliations:** 1 Department of Radiology, NorthShore University HealthSystem, Evanston, IL 60201, USA; 2 Department of Biomedical Engineering, Northwestern University, Evanston, IL 60208, USA; 3 Department of Pediatrics, NorthShore University HealthSystem, Evanston, IL 60201, USA; 4 Department of Anesthesiology, NorthShore University HealthSystem, Evanston, IL 60201, USA; 5 Pritzker School of Medicine, University of Chicago, Chicago, IL 60637, USA

**Keywords:** ephaptic coupling, burst-suppression, seizures

## Abstract

Electric fields are now considered a major mechanism of epileptiform activity. However, it is not clear if another electrophysiological phenomenon, burst suppression, utilizes the same mechanism for its bursting phase. Thus, the purpose of this study was to compare the role of ephaptic coupling—the recruitment of neighboring cells via electric fields—in generating bursts in epilepsy and burst suppression. We used local injections of the GABA-antagonist picrotoxin to elicit epileptic activity and a general anesthetic, sevoflurane, to elicit burst suppression in rabbits. Then, we applied an established computational model of pyramidal cells to simulate neuronal activity in a 3-dimensional grid, with an additional parameter to trigger a suppression phase based on extra-cellular calcium dynamics. We discovered that coupling via electric fields was sufficient to produce bursting in scenarios where inhibitory control of excitatory neurons was sufficiently low. Under anesthesia conditions, bursting occurs with lower neuronal recruitment in comparison to seizures. Our model predicts that due to the effect of electric fields, the magnitude of bursts during seizures should be roughly 2–3 times the magnitude of bursts that occur during burst suppression, which is consistent with our in vivo experimental results. The resulting difference in magnitude between bursts during anesthesia and epileptiform bursts reflects the strength of the electric field effect, which suggests that burst suppression and epilepsy share the same ephaptic coupling mechanism.

## 1. Introduction

Epilepsy is a prevalent neurological disorder characterized by recurrent seizures that affect over 70 million people worldwide and significantly impact their daily lives. Seizures manifest as impaired consciousness and convulsions, due to disturbed neuronal networks rather than local abnormalities [1] (for a full operational classification of the common types of seizures from the International League Against Epilepsy (ILAE), see [2]). Epilepsy is associated with various neurological comorbidities such as stroke, dementia, migraines, brain tumors, and non-neurological conditions such as heart disease, hypertension, and chronic obstructive pulmonary disease [3].

The abnormal electrical activity in the brain that causes seizures stems from factors such as genetic mutations, brain injury, and infections. Epileptic activity occurs when the balance between excitatory and inhibitory signaling tilts heavily towards excitation, often caused by deficient inhibitory interneurons in the cerebral cortex. Synchronized excitatory potentials overwhelm the inhibitory network, leading to hypersynchronization. Current pharmacological treatments for epilepsy only prove effective in approximately 60–70% of cases [4] and most commonly rely on the use of benzodiazepines, barbiturates, and other anticonvulsants (for a review, see [5]).

In addition to altered neurotransmitter signaling, electric fields are known to play an important role in the mechanisms of epilepsy by influencing the synchronization of neuronal activity in the brain. Electric fields are thought to substantially (or perhaps even critically) contribute to this synchronization of neuronal activity by facilitating the spread of electrical signals across large populations of neurons [6,7].

Burst suppression is another brain electrical pattern characterized by high-frequency bursts followed by periods of silence. This pattern often looks similar to mild local epileptic activity [8] and can be induced in the brain by various means, including anesthesia, hypothermia, or certain medications. Burst suppression under general anesthesia has received great attention in recent years because of its potential danger to brain health. Specifically, burst suppression was associated with anesthesia-related adverse effects [9,10,11]. These effects include confusion, agitation, somnolence, dizziness, and drowsiness. Data suggest that up to 87% of the elderly population experience postoperative cognitive dysfunction [12], and approximately 36% of adult patients experience delayed recovery from anesthesia, including postoperative nausea and vomiting [13]. The severity of these effects is positively correlated with the duration of burst suppression [9,10,11]. These symptoms can have a significant impact on patients’ well-being, prolong hospitalization, and increase healthcare costs. The mechanisms behind burst suppression remain unclear, and its similarity to epileptic activity at the level of multi-units [8] raises questions about the role of electric fields in this phenomenon.

General anesthesia is known to significantly reduce neuronal activity, even without inducing burst suppression. Studies have shown that general anesthesia can cause a drop in neuronal activity to 50% or less than the baseline awake level, depending on the dose [14,15,16,17]. This significant decrease in neuronal activity plays a fundamental role in inducing the desired state of unconsciousness during anesthesia. Interestingly, despite the overall reduction in neuronal activity, the total level of inhibition actually decreases compared to the awake state, which may seem counterintuitive. This phenomenon is attributed to a decrease in the firing rate of interneurons, which serve as the primary source of the inhibitory neurotransmitter GABA in the cerebral cortex [14]. The reduction in interneuron firing rate is proportional to the overall decrease in neuronal activity, leading to a decline in the release of GABA and contributing to the overall decrease in inhibition. Even though inhibitory signaling decreases, the effects of anesthesia can still be achieved through a severe reduction in excitatory signaling [14]. Understanding the mechanisms behind burst suppression opens up new possibilities for pharmacological interventions to prevent or manage this phenomenon. The identification of these mechanisms not only paves the way for better management of burst suppression but also provides valuable information about the complex interactions in the brain during anesthesia.

In this study, we aim to investigate the potential shared ephaptic mechanisms between epileptic activity and burst suppression. We hypothesize that electric fields play a significant role in both phenomena, impacting the expected reduction in inhibition. Our approach involves a combination of experimental data and a previously published computational model of electric fields. To induce epileptic activity, we used the GABAA-antagonist picrotoxin (PTX) in awake rabbits, focusing on localized seizure activity rather than a comprehensive epilepsy model. Furthermore, to induce burst suppression, we employed the general anesthetic sevoflurane (SVF). Despite the differences in their specific effects, both drugs induce a shift in the excitatory/inhibitory balance towards excitation, leading to comparable impacts on electric fields. However, in the case of sevoflurane, the electric field effect is relatively weaker due to its milder shift in the excitatory/inhibitory balance. Consequently, the reduction in inhibition during sevoflurane-induced burst suppression is estimated to be relatively milder compared to our model of local epileptic activity, primarily resulting from a decrease in the firing of interneurons. Our computational approach was based on the novel model by Shivacharan et al. [6]. Our findings suggest that both epileptic activity and burst suppression share similar mechanisms involving electric fields, though the impact on neuronal activity during burst suppression is relatively smaller.

## 2. Materials and Methods

### 2.1. Animal Preparation

Female pigmented Dutch-belted rabbits (2–3 kg, 6–12 months of age) were used in these experiments, in accordance with the National Institutes of Health guidelines and with the approval of NorthShore University Health System’s Institutional Animal Care and Use Committee. Two groups of animals were used for the experiments. In the first group (N = 5), the dynamics of resting-state neuronal activity after injections of PTX were studied. In the second group (N = 5), resting-state neuronal activity was studied after the administration of SVF. The total number of rabbits was 10. We generally followed our previously described methods [18]. Animals were anesthetized with a mixture of ketamine (50 mg/kg) and xylazine (10 mg/kg), an incision was made in the scalp, and the bone was exposed on the top of the skull. A light-weight head restraining device containing four nylon bolts was implanted on the top of the skull. This head bolt was used to secure the radiofrequency (RF) coil and the animal’s head in the same position in order to obtain reproducible slice positioning among subjects, as previously described [18]. For neuronal recording, the assembly was implanted, consisting of a bundle of four 25 µm diameter gold-silver alloy microwires with formvar insulation (California Fine Wire, Grover Beach, CA, USA) inside a silica tube (Polymicro Technologies, Phoenix, AZ, USA). The electrode materials were chosen based on our previous evaluation to minimize susceptibility to artifacts in MR images. These electrodes terminated at different levels within a distance of 100 µm and were attached to a permanently implanted, custom-made nylon microdrive that permitted vertical adjustments of its position. The microwires were connected to a small 6-pin connector that was embedded in dental acrylic. A 150 µm silver wire was placed between the skull and dura mater to serve as the reference electrode. A 200 µm silica injection cannula was attached to the microdrive. During implantation surgery, lambda was positioned 1.5 mm below the bregma, and the stereotaxic coordinates were as follows: anterior-posterior was 2 mm dorsal to the bregma, medial-lateral was 6 mm from the midline, and dorsal-ventral was under visual control relative to the surface of the cerebral cortex. Later, the electrodes were advanced to layer IV of the cerebral cortex. One week after the implantation surgery, each subject (N = 10) in both groups was habituated to the cloth bag and environment for resting-state electrophysiology recording.

We used optogenetic stimulation to determine the stacking factor during a normal awake state by measuring the change in the strength of the local field potential (LFP) signal from two separate electrodes at set distances from the source of stimulation. We followed our previously published approach for optogenetic stimulation [19]. The adeno-associated viral vector pAAV-CaMKIIα::hChR2(H134R)-EYFP (2 × 10^12^ particles/mL) was obtained from the University of North Carolina Vector Core (Chapel Hill, NC, USA). Prior to virus injection, rabbits underwent optogenetic stimulation in control experiments to ensure that the optical stimulation itself did not produce changes in electrophysiological and BOLD responses. Concentrated virus (2 µL/site) was injected through implanted cannulae at 4 sites 300 µm apart to obtain sufficient coverage of expression within the barrel cortex. Injections were delivered through a silica tube/needle inserted in an implanted cannula and connected to a 50-μL Hamilton syringe using transparent Tygon tubing. Animals were allowed 1 month following injection to reach full ChR2 expression prior to experiments. Optical stimulation consisted of blue (473 nm) light generated by a DPSS laser system (Optotronics, Mead, CO, USA) delivered through a 200 µm fiber at 50 Hz.

To detect the location of electrodes, imaging was performed using a 9.4 T imaging spectrometer (BioSpec 94/30USR, Bruker, Billerica, MA, USA) operating at a proton frequency of 400 MHz. This system was equipped with an Oxford horizontal magnet and an Acustar actively shielded gradient coil assembly with a clear bore of 15 cm. A flat, circular surface coil (20 mm in diameter) was used for RF transmission and reception. High-resolution anatomical images (512 × 512 matrix, 48 mm × 48 mm FOV, equivalent to 94 µm × 94 µm in-plane resolution) were obtained using a multi-slice gradient echo sequence (1.0 mm slice thickness; TR, 1.5 s; TE, 20 ms, NA = 8). The examples of the images were reported previously [20] (Figure S1).

### 2.2. Electrophysiological Recording and Microinjections

The electrophysiological signals from the microwires were fed through a miniature preamplifier to a multi-channel differential amplifier system (Neuralynx, Inc., Bozeman, MT, USA). The signals were amplified, band-pass filtered (0.3–3 kHz for single units), and digitized (32 kHz/channel) using a Neuralynx data acquisition system. Electrophysiological signals from neuronal activity were analyzed after the removal of blocks of strong interference signals induced by gradient pulses. These blocks were detected by thresholding, followed by one-dimensional mathematical morphology [21] processing based on erosion and dilation functions. To capture the initial neuronal activity without the interfering signals from the gradients, the stimulus onset was delayed for 150 ms from the MR acquisition triggering pulse. Subsequently, unit discrimination was performed offline using threshold detection, followed by a cluster analysis of scatter plots of time and amplitude distances between the peak and valley of individual action potential wave shapes. The discriminated data were processed using Neuralynx and custom software written in Matlab and Visual Basic. Peri-event histograms were constructed for each unit and experiment before and after the PTX injection. In each histogram, the baseline firing rate and the magnitude of excitatory changes were computed. A baseline firing rate was calculated for all units. The mean single-unit activity was calculated only for units that exhibited an excitatory response. Individual normalized cell histograms (spike frequency) were pooled together for each cell type and period of time to construct average population histograms. Single units were converted to 1 Hz to smooth out the deviation from the baseline. Picrotoxin (248 µM) was dissolved in artificial cerebro-spinal fluid (ACSF) for injection. This concentration of PTX was based on our preliminary studies, which showed that under this concentration there were no actual seizures. All injections (1 μL volume) were delivered through a silica tube/needle (190 μm OD and 100 μm ID, Polymicro Technologies, LLC, Phoenix, AZ, USA) and connected to a Hamilton syringe using transparent Tygon tubing. Equal volumes of vehicle (ACSF) were injected into the same rabbits in a randomized order (i.e., either before or after PTX injection) following the same procedure in order to control for potential injection-related effects. Neuronal activity was monitored continuously to ensure that the volume effect was minimized [20]. Based on our previous data [8,20], 15 min is sufficient to reach the stability of the injection effect, avoid any potential artifacts related to the volume effect of the injection, and allow PTX to diffuse throughout the whisker barrel cortex. Bursts typically appear 5–10 min after the injection.

During the anesthesia experiments, both single-unit activity and local field potential (LFP) were continuously recorded. The recordings commenced with a 5-min period in the awake state. Subsequently, the rabbits were anesthetized via mask with sevoflurane (AbbVie Inc., North Chicago, IL, USA) at a level of 1 minimum alveolar concentration (MAC) using calibrated vaporizers (Drager Vapor 19.1). In rabbits, 1 MAC of sevoflurane typically corresponds to approximately 4% sevoflurane concentration [22]. Throughout the anesthesia, we monitored spontaneous respiration using a pressure pad/respiration transducer (TSD110) from Biopac Systems, Inc., Goleta, CA, USA. For analysis, we specifically selected experiments that exhibited burst suppression. In each experiment, we meticulously chose one electrode for analysis based on the quality of the recording.

### 2.3. Computational Model

#### 2.3.1. Two-Compartment Model

To model the dynamics of neuron activity, we applied the two compartment soma-dendrite model introduced by Pinsky et al. [23]. The transmembrane potential is governed by the equations

(1) $$C_{m}{V_{s}}^{\prime }=-I_{leak}\left(V_{s}\right)-I_{Na}\left(V_{s},h\right)-I_{K-DR}\left(V_{s},\;n\right)+g_{c}\left(V_{d}-V_{s}+V_{out}\right)+I_{s}$$

(2) $$C_{m}{V_{d}}^{\prime }=-I_{leak}\left(V_{d}\right)-I_{Ca}\left(V_{d},s\right)-I_{K-AHP}\left(V_{d},\;q\right)-I_{K-C}\left(V_{d},\;Ca,c\right)-g_{c}\left(V_{d}-V_{s}+V_{out}\right)+I_{d}$$

where $V_{s}$ and $V_{d}$ (in mV) are the deviations of transmembrane potential from the resting potential for the soma and dendrite, respectively. For this study, the resting potential is 0 mV. The injected current into the soma and dendrite, respectively, is denoted by $I_{s}$ and $I_{d}$. The soma compartment contains an inward sodium current $I_{Na}$ and outward delayed-rectifier potassium current $I_{K-DR}$, while the dendrite component contains the inward calcium current $I_{Ca}$, and two outward potassium currents, $I_{K-C}$ which is voltage dependent but proportional to the saturation of calcium, and $I_{K-AHP}$ which is dependent on intracellular calcium concentrations. The leak current for both compartments is denoted by $I_{leak}$. All currents are in units of $\mu A/{cm}^{2}$. The variable $g_{c}$ is a conductance term (unit of $mS/{cm}^{2}$) denoting the strength of coupling between the soma and dendrite component, with $V_{out}\;:=V_{out}^{d}-V_{out}^{s}$ where $V_{out}^{d}$ represents the extracellular potential at the dendrite and $V_{out}^{s}$ the extracellular potential at the soma [24]. For this study, we set $g_{c}=1$ $mS/{cm}^{2}$. The conductance $g_{c}$ times the difference in transmembrane plus the $V_{out}$ term is introduced to allow for both electronic coupling between the two compartments and the electric field effect via the difference in extracellular potential between the two compartments. The equations for the ion channels are:

$$I_{leak}\left(V_{s}\right)=g_{L}(V_{s}-V_{L})$$

$$I_{leak}\left(V_{d}\right)=g_{L}(V_{d}-V_{L})$$

$$I_{Na}=g_{Na}m_{\infty }^{2}\left(V_{s}\right)h(V_{s}-V_{Na})$$

$$I_{K-DR}=g_{K-DR}n(V_{s}-V_{K})$$

$$I_{Ca}=g_{Ca}s^{2}(V_{d}-V_{Ca})$$

$$I_{K-C}=g_{K-C}c\chi \left(Ca\right)\left(V_{d}-V_{K}\right)$$

$$I_{K-AHP}=g_{K-AHP}q(V_{d}-V_{K})$$

with $\chi \left(Ca\right)=min(\frac{Ca}{250},1)$

The kinetic equations for each gating variable are given by

$$h^{\prime }=(h_{\infty }\left(V_{s}\right)-h)/\tau (V_{s})$$

$$n^{\prime }=(n_{\infty }\left(V_{s}\right)-n)/\tau (V_{s})$$

$$s^{\prime }=(s_{\infty }\left(V_{d}\right)-s)/\tau (V_{d})$$

$$c^{\prime }=(c_{\infty }\left(V_{d}\right)-c)/\tau (V_{d})$$

$$q^{\prime }=(q_{\infty }\left(Ca\right)-q)/\tau (Ca)$$

The equation for Ca dynamics in the dendritic compartment is given by

$$Ca^{\prime }=-0.13I_{Ca}-0.075Ca$$

The steady state values for each gating variable are in the form $y_{\infty }=\alpha_{y}/(\alpha_{y}+\beta_{y})$, while the time constants are in the form $\tau =1/(\alpha_{y}+\beta_{y})$ for y=h,n,s,c,q. The functions $\alpha_{y}$ and $\beta_{y}$ for all values of y are

$$\alpha_{m}=\frac{0.32\left(13.1-V_{s}\right)}{\exp\left(\frac{13.1-V_{s}}{4}\right)-1}$$

$$\beta_{m}=\frac{0.28\left(V_{s}-40.1\right)}{\exp\left(\frac{V_{s}-40.1}{5}\right)-1}$$

$$\alpha_{n}=\frac{0.016\left(35.1-V_{s}\right)}{\exp\left(\frac{35.1-V_{s}}{5}\right)-1}$$

$$\beta_{n}=0.25\exp\left(0.5-0.025V_{s}\right)$$

$$\alpha_{h}=0.128\exp\left(\frac{17-V_{s}}{18}\right)$$

$$\beta_{h}=\frac{4}{1+\exp\left(\frac{40-V_{s}}{5}\right)}$$

$$\alpha_{s}=\frac{1.6}{1+\exp\left(-0.072(V_{d}-65)\right)}$$

$$\beta_{s}=\frac{0.2\left(V_{d}-51.1\right)}{\exp\left(\frac{V_{d}-51.1}{5}\right)-1}$$

$$\alpha_{c}=\frac{\exp\left(\frac{V_{d}-10}{11}\right)-\exp\left(\frac{V_{d}-6.5}{27}\right)}{18.975}\;V_{d}\leq 50$$

$$\alpha_{c}=2exp(\frac{6.5-V_{d}}{27})\;V_{d}>50$$

$$\beta_{c}=2\exp\left(\frac{6.5-V_{d}}{27}\right)-\alpha_{c}\;V_{d}\leq 50$$

$$\beta_{c}=0\;V_{d}\leq 50$$

$$\alpha_{q}=\min\left(\left(0.00002\right)Ca,\;0.01\right)$$

$$\beta_{q}=0.001$$

For this study, we used the parameter values set by Pinsky et al., with maximal conductances (in $mS/{cm}^{2})$ $g_{L}=0.1$, $g_{Na}=30$, $g_{K-DR}=15$, $g_{Ca}=10$, $g_{K-C}=15$, $g_{K-AHP}=0.8$. The reversal potentials (in mV) are $V_{L}=0$, $V_{Na}=120$, $V_{Ca}=140$, $V_{K}=-15$.

#### 2.3.2. Computational Network

The network consists of a 3728 μm (x-axis) × 250 μm (y-axis) × 360 μm (z-axis) grid. The x-axis consists of 201 grid points, the y-axis consists of 2 grid points, and the z-axis consists of 19 grid points. The grid points along the X-Z plane correspond to individual neurons, while the grid points along the y-axis correspond to either the soma or the dendrite. For example, for some (i,j,k) in the 3D grid, j = 1 would correspond to the soma, and j = 2 would correspond to the dendrite for neuron (i,k) in the X-Z plane. Each grid point corresponds to a separate neuron, thus creating an array of parallel, back-to-back neurons.

For the soma, we vary the injected current, $I_{s}$, between −0.5 and −0.4 (generated randomly from a uniform distribution), which corresponds to a neuronal firing rate between 0–4 Hz, while the injected current for dendrites, $I_{d}$, is set to 0. The purpose of this setup is to stimulate the spontaneous firing rate of pyramidal cells, which would accurately reflect what is seen in experimentally recorded LFP.

The coupled equations are iterated over the grid using a forward Euler numerical method with a time step of 0.1 (in units of microseconds).

The suppression phase was simulated by injecting an additional current of −0.8 $\mu A/{cm}^{2}$ whenever the sum of all the calcium levels (Ca) in the cell exceeded 2 (A.u.). The sum of the intra-cellular calcium levels was used as a proxy for the level of extracellular calcium. The current was applied for 500 ms when the stacking factor was 15, and 750 ms when the stacking factor was 30.

#### 2.3.3. Electric Field Coupling

In accordance with [6], we calculate the extra-cellular potential at a recording site based on a weighted summation of all transmembrane currents scaled by distance relative to the site. The equation for the extracellular potential at grid point $\left(i,j,k\right)$ is given by

$$V_{ijk}=SF*\frac{\rho }{4\pi }\sum_{z\;\epsilon \;G}\frac{I_{z\;}}{r_{z\;}}$$

where SF is the stacking factor (in A.u.), ρ is the extracellular resistivity (in Ω cm), and $V_{ijk}$ is the extracellular potential (in mV) at the grid point located at $\left(i,j,k\right)$. The value $z:(i^{\prime },j^{\prime },k^{\prime })$ is a 3-element vector corresponding to a grid point in G, which is the set of all points in the total grid such that $k^{\prime }\neq k$. Thus, $I_{z}$ is the transmembrane current (in mA) at grid point $\left(i^{\prime },j^{\prime },k^{\prime }\right)$ and $r_{z\;}$(in cm) is the Euclidian distance between (i,j,k) and $\left(i^{\prime },j^{\prime },k^{\prime }\right)$. Let i,k be fixed values, and then in accordance with our two-compartment model $V_{i1k}$ would correspond to $V_{out}^{s}$ and $V_{i2k}$ would correspond to $V_{out}^{d}$, thus constituting our electric field. For this study, we set the extracellular resistivity, ρ, to 380 Ωcm.

The stacking factor represents the number of actively firing neurons located within the grid point. For example, a stacking factor of 1 would imply that only one active neuron in the entire area occupied the grid point. Varying the stacking factor allows both the neuronal density of a specific brain region and the excitability of the neuronal network to be accounted for. For this study, we assume that for the somatosensory cortex, a stacking factor of 4 corresponds to 100% functioning of the inhibitory system, a stacking factor of 15 corresponds to 50% functioning of the inhibitory system, and a stacking factor of 30 corresponds to 0% functioning of the inhibitory system. Increasing the stacking factor will in turn increase the magnitude of the electric fields generated, which in turn will promote seizure-like activity while simultaneously increasing the magnitude of LFP fluctuations.

The density of neurons in the somatosensory cortex can reach up to 90,000 neurons per mm^2^, according to [25]. The horizontal axis (the X-Z plane) of our computational grid consists of grid points separated by 18.64 microns along the x-axis and 20 microns along the z-axis, giving an approximate surface area of about 3.73 × 10^−4^ mm^2^ corresponding to each grid point. Thus, each grid point contains approximately 33 active neurons. Assuming roughly 10% of these neurons are inhibitory, this means that we expect about 30 pyramidal cells. During the awake resting state, the excitatory/inhibitory balance leads to sparsity in the activity of neurons, such that roughly only 10% of neurons are actively firing in the network [26]. This led us to use a stacking factor of 4 to simulate the electric field effect during the awake, resting state. For 50% functioning of the inhibitory system, which represents burst suppression, we set the stacking factor to 15. We attributed 50% of the reduction in inhibition to sevoflurane due to its effect on the firing rate of interneurons while simultaneously potentiating the tonic GABA currents [14,27]. For 0% functioning of the inhibitory system, which represents the blocking of GABAA receptors, we set the stacking factor to 30.

To more accurately measure the stacking factor during the resting state, dependent on the rabbit, experimental setup, and electrode placement, we set up an optogenetic experiment to measure the decay of the LFP signal in relation to distance. Our special electrode setup had two electrodes, 100 microns apart, at a distance of 200 microns from the site of optogenetic stimulation. Thus, we could compare the difference in peak-to-peak magnitude during each stimulation event between the LFP in electrode 1 and electrode 2. To ensure a smoother, more pronounced peak, we smoothed the processed 200 Hz data using averaging with an overlapping window of 10 time points (0.5 s). To compare with the computational results, we simulated stimulation via an injected current of 0.5 mA to the middle 20 neurons in our 3D grid. The LFP for the resulting simulation was calculated 200 microns away and 300 microns away from the stimulation site in the direction of the x-axis. Smoothing was also applied to the computational LFP data via Gaussian smoothing with a window of 10 time points (0.5 s). From there, using MSE between the vector of electrode values from the experimental recordings and the electrode values from the computational simulation, we found the stacking factor, which closely aligned with our experimental recordings. In this manner, we determined that a stacking factor of 5.4 was the closest fit for our rabbits and our electrode setup, specifically.

#### 2.3.4. Statistical Analysis

Our data consists of five experiments for SVF and five experiments for PTX. There were 1486 burst events for SVF and 1176 burst events for PTX. Eight different LFP metrics were calculated to compare experimental SVF and PTX data: Mid-frequency, median frequency, average peak amplitude, average distance between peaks, average LFP peak rate, delta power, theta power, and gamma power. The metrics were calculated in bins of 0.5 s (resulting in metric data with a sampling rate of 2 Hz) and between 5–25 min for SVF data and 20–40 min for PTX data. Mid-frequency was calculated as the mean frequency value from the power spectral density (PSD) of LFP within each 0.5 s bin of LFP data, and median frequency was calculated as the median frequency value of the PSD from each 0.5 s bin. Peaks were defined as LFP values that reached a local peak exceeding one standard deviation (i.e., a peak corresponding to burst behavior). The average peak amplitude was calculated as the average peak value for each 0.5 s bin. The average distance between peaks was the average number of time points between each peak for each 0.5 s bin. Delta, theta, and gamma power were calculated as the density for the 0–4 Hz, 4–7 Hz, and 36–100 Hz frequency ranges of LFP. Statistical significance was assumed at a *p* value < 0.05. The Kolmogorov-Smirnov test was used to verify the normal distribution.

To determine which metrics to run for a comparison analysis between PTX and SVF, principal components (PCA) were calculated for each experiment, with the aforementioned metrics used as the 8 features for each experiment. Using only the loadings from the first principal component, the features with the three highest absolute loading values are selected.

The metrics that satisfy the PCA criteria are then averaged for each experiment, and Welch’s t-test is run to compare if the selected metric is significantly different between the SVF and PTX experiments.

## 3. Results

**Figure 1:**
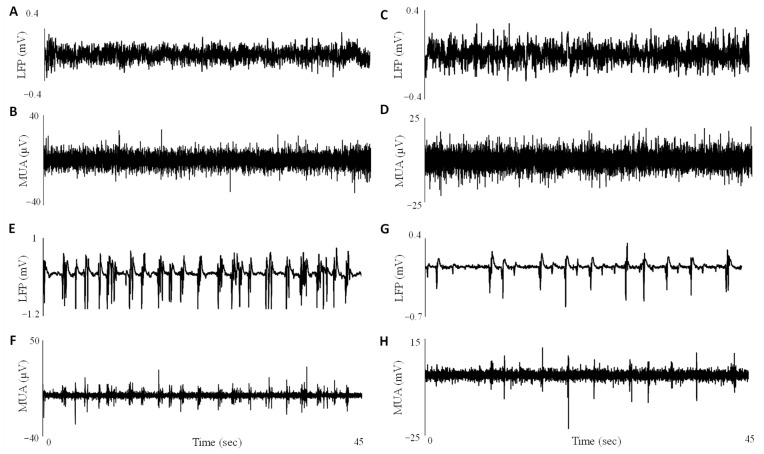
A comparison between the awake resting state and burst suppression electrophysiology induced by sevoflurane. (**A**,**B**) In the awake resting state, both local field potentials (LFP) (**A**) and normalized multi-unit activity (MUA) (**B**) showed no discernible pattern, with LFP exhibiting low magnitudes. (**C**,**D**) However, under 0.5 MAC of sevoflurane, slow dynamics emerged in LFP (**C**), while this was not visually reflected in MUA (**D**). (**E**,**F**) As the sevoflurane concentration increased to 1 MAC, the characteristic burst suppression pattern became evident in both LFP (**E**) and MUA (**F**). Interestingly, MUA closely followed the dynamics of LFP, with large spikes in MUA corresponding to bursts in LFP. (**G**,**H**) Additional examples of burst suppression are shown in panels (**G**,**H**) for LFP and MUA, respectively, under 1.5 MAC. Both examples (**E**,**G**) exhibit burst suppression; however, notable differences exist in the form of their burst suppression patterns and the duration of the suppression phases. The presence of diverse burst suppression patterns suggests a probabilistic perspective on the timing of these events, which may depend on the level of glutamatergic excitatory activity that becomes more diminished at higher sevoflurane concentrations.

We examined electrophysiological activity (LFP and MUA) in the resting state in comparison to the activity under the administration of sevoflurane. In the awake resting state, LFP (Figure 1A) tends to stay below 0.2 mV and has no visible relationship with MUA (Figure 1B). At 0.5 MAC of sevoflurane, the magnitude of LFP fluctuations (Figure 1C) nearly doubles in comparison to the awake state; however, the dynamics of MUA (Figure 1D) are similar to those of the awake state in terms of the absence of any distinguishable pattern. Increasing the concentration of sevoflurane to 1 MAC introduced the burst-suppression pattern visible in LFP (Figure 1E), with the burst phase accompanied by spikes in MUA (Figure 1F). Another example with 1 MAC of sevoflurane also exhibits burst suppression; however, with more regular bursting and shorter suppression phases (Figure 1G,H for LFP and MUA, respectively). Before and during anesthesia, the respiration rate was recorded (59.4 ± 4.9 and 39.6 ± 9.1 cycles per minute (cpm), respectively). The heart rate was recorded only during anesthesia and was 66.4 ± 23.9 cpm.

**Figure 2:**
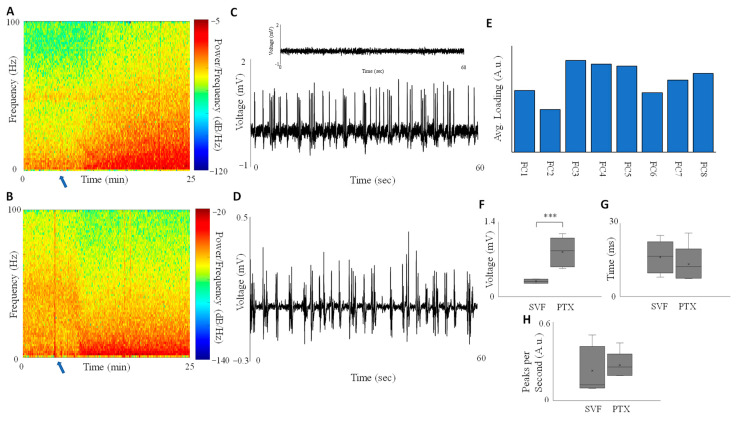
Comparison of LFP magnitudes between seizures and burst suppression. (**A**) LFP spectrograms are shown for experiments involving injection of the GABA-antagonist picrotoxin (PTX) at 5 min and (**B**) exposure to sevoflurane (SVF) at 5 min (blue arrow). The spectrograms reveal an increase in power within the delta-range frequency, which is indicative of seizure-like activity. (**C**) Notably, the magnitude of the seizure bursts during PTX injection is significantly higher compared to the burst suppression observed during sevoflurane exposure (**D**). The insert represents LFP before PTX injection. (**E**) PCA analysis shows that the metrics FC3, FC4, and FC5 (average spike amplitude, average peak to peak distance, and average spike rate, respectively) have the highest three loadings. The labels FC1, FC2, FC3, FC4, FC5, FC6, FC7, and FC8 correspond to mid-frequency, medium frequency, average spike amplitude, average peak-to-peak distance, average spike rate, gamma band power, delta band power, and theta band power, respectively. (**F**) PTX has a statistically higher average spike amplitude than SVF after running an unpaired *t*-test over all experiments (*** *p* < 0.005). (**G**) Peak-to-peak distances and (**H**) spike rates show no statistical difference between PTX and SVF.

Using picrotoxin (PTX) injections and exposure to sevoflurane (SVF) to induce seizures and burst suppression, respectively, we compared the characteristics of both events via LFP recordings. The LFP spectrogram for an experiment where PTX was injected at 15 min (Figure 2A) shows an increase in power in nearly all frequency bands from the range 0–100 Hz roughly 5 min after injection, with the largest increase being in the lower frequency (delta) band, typical for seizure-like events. The corresponding time series of LFP band power are shown in Figure S2. Administrating SVF at 5 min (Figure 2B), there is also a sizable increase in the delta band 5 min after administration, without an increase in higher frequency ranges. An illustrative one-minute LFP series for the PTX injection (Figure 2C) and SVF (Figure 2D) shows that seizures induced by PTX occur at a larger magnitude (up to 1.5 mV or even bigger depending on the PTX concentration); however, the frequency of the bursts remains similar. This difference in magnitudes is shown to be statistically significant (*p* < 0.005) using Welch’s *t*-test over all experiments (Figure 2E).

**Figure 3:**
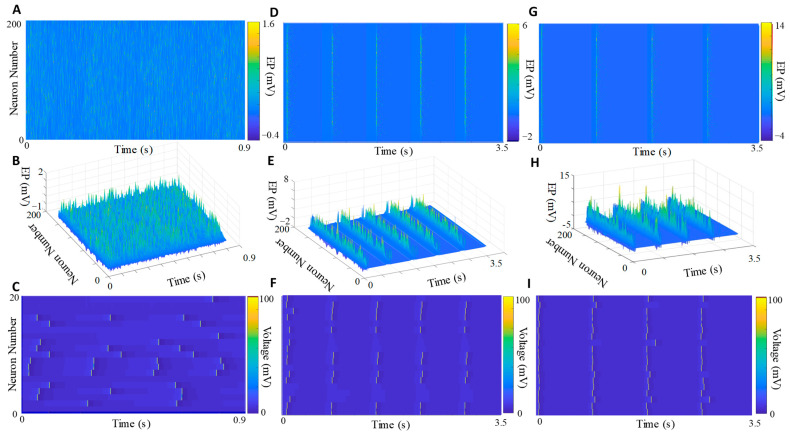
Decreased inhibition and the formation of seizure-like activity via electric fields. (**A**,**B**) At 100% inhibition, representing normal resting state activity, extracellular potentials (EP) near the soma typically remain low, rarely exceeding 1.5 mV. (**C**) Neuronal firing occurs at random rates without any synchrony observed among the population. (**D**,**E**) When inhibition is reduced to 50%, there is a notable increase in the magnitude of EP compared to the 100% inhibition state. (**F**) Additionally, there is an increased synchronization of action potentials, occurring alongside random firing patterns. (**G**,**H**) At 0% inhibition, EP can reach as high as 15 mV near the soma (**I**), and there is near-total synchrony in the firing of all neurons within the population. Note that for panels (**C**,**F**,**I**), only the middle 20 neurons of the grid are shown for better visibility.

To further examine the driving force behind how SVF and PTX can affect the LFP signal, we simulated the firing patterns of a two-compartment soma-dendrite neuronal network over a 3-dimensional grid, using electric fields to determine the effect on extra-cellular potentials and synchronization. Under normal firing conditions, which we term “100% inhibition,” extracellular potential stays below 1.5 mV and there is no discernable synchronization among the population of neurons (Figure 3A–C). At 50% inhibition, which we take as a representative condition under SVF, there is already an increase in the voltage of extra-cellular potentials along with some partial synchronization among the population (Figure 3D–F). However, these characteristics represent only a fraction of the effect produced under 0% inhibition, where we consistently observe near-total synchronized firing. As a result, there is a substantial increase in extracellular potentials near dendrites (Figure 3G–I), indicating that the level of inhibition influences neurons to exhibit seizure-like behavior on a gradient basis via the electric field effect. Moreover, we demonstrate that removing electric field coupling under 0% inhibitory control disrupts neuronal synchronization (Figure S3). Additionally, we demonstrate the tuning of the stacking factor to match the optogenetic stimulus with the computational model (Figure S4).

**Figure 4:**
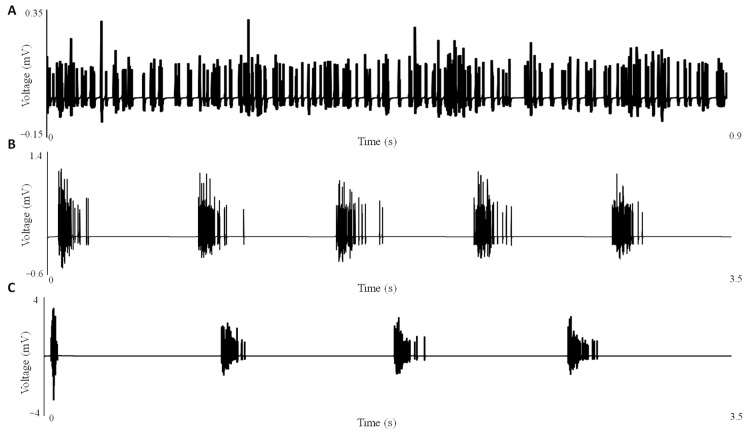
Local field potentials near the soma under different levels of inhibition. (**A**) The LFP series for 100% inhibition, (**B**) 50% inhibition, and (**C**) 0% inhibition demonstrate the progressive increase in LFP magnitude and the emergence of bursting activity driven by the electric field effect.

Figure 4A–C illustrate the effect of inhibition on the LFP signal, which is located near the soma. As inhibition decreases, there is a corresponding increase in the magnitude of the LFP fluctuations due to the stacking factor, along with the formation of synchronized clusters due to the electric field effect.

## 4. Discussion

In this study, we have presented evidence supporting ephaptic interactions as a potential mechanism underlying anesthesia-induced burst suppression. By combining ephaptic coupling with inhibition due to extracellular calcium dynamics, we propose a non-synaptic mechanism that provides a common pathway to explain the occurrence of burst suppression under various scenarios. While previous studies have implicated ephaptic coupling in burst generation during epilepsy [6,28,29], our analysis, which is generally based on a validated model for epilepsy [6], suggests that a similar mechanism may be at play during anesthesia-induced burst suppression. Figure 1 shows the varying dynamics of LFP and MUA during the resting state and at 0.5 MAC, 1 MAC, and 1.5 MAC of sevoflurane. Although 0.5 MAC of sevoflurane does not produce burst suppression, it can increase the magnitude of fluctuations by twofold. However, there is no change in the dynamics of MUA. The increase in the magnitude of fluctuations in LFP is consistent with previous studies that observed a rise in the delta power component of EEG during sevoflurane [30]. Increasing the concentration of sevoflurane to 1 MAC introduces the burst suppression pattern; however, the form of the pattern may vary from experiment to experiment. In one example in Panels E and F (Figure 1), the suppression phase between bursts can vary, often in terms of seconds, while in the examples in Panels G and H, there is more regularity in the time between bursts.

The varying lengths between bursts suggest that bursts may occur in a probabilistic manner. Previous studies have utilized Bayesian-based probability models to assess the likelihood of being in the suppression phase of burst suppression [31]. This aligns well with our own observations, where we noticed that increased concentrations of sevoflurane led to longer durations of the suppression phase (Figure 1). In our model, we describe the suppression phase as a function of extracellular calcium dynamics and the probabilistic nature of bursts arising from spontaneous neuronal firing. As sevoflurane increases (Figure 1G,H), it further suppresses glutamatergic activity [32], thereby leading to the expected extension of the suppression phase.

We compared local field potentials (LFP) recorded under the influence of the GABA antagonist picrotoxin and the volatile anesthetic sevoflurane (Figure 2). Our spectrogram analysis revealed distinct differences in the power spectra of LFP between the two conditions. Specifically, PTX administration resulted in an increase in power across most frequency bands, with a pronounced enhancement in the lower frequency ranges (<20 Hz). These findings are consistent with previous studies showing that PTX can increase lower frequency power in the LFP spectra, even at lower concentrations [33]. Moreover, an increase in gamma band EEG activity has been reported at higher PTX doses [34]. Our results also align with prior observations of increased delta band dominance during sevoflurane anesthesia [30].

The resemblance in the behavior of lower-frequency power spectra between PTX and sevoflurane suggests a shared underlying mechanism involving the synchronization of neuronal firing rates. This synchronization has been known to impact changes in delta band power [35] and can be achieved in in vitro preparations [36]. Furthermore, we observed that the bursts induced by local injections of PTX generally exhibited larger magnitudes compared to those under sevoflurane, despite following a similar bursting pattern. Our statistical analysis confirmed that the magnitude of LFP changes under PTX was significantly greater than that observed under sevoflurane across all experiments. The difference in LFP magnitude between epileptic activity and burst suppression may reflect the level of neuronal recruitment through electric fields, as demonstrated in our model (Figure 3). When assuming 100% functioning of the inhibitory system, the extracellular potentials near the soma remained below 1 mV, indicating a lack of synchronization and normal spontaneous awake activity.

Thus, we employed a computational model to simulate a scenario where the level of inhibition was reduced by 50% in conjunction with a decrease in the firing of excitatory neurons. This modeling approach allowed us to investigate how altering the inhibitory dynamics of the network would affect its overall state. Interestingly, we observed that this modification had a significant impact, increasing the magnitude of extracellular potentials and leading to a stronger electric field effect. This increased electric field effect resulted in notable changes in network activity and the emergence of synchronized burst patterns. These findings highlight the intricate relationship between inhibition, excitatory neuronal firing, and their sensitivity to electric fields. In this scenario, bursts began to emerge, accompanied by a significant increase in extracellular potentials (reaching up to 5–6 mV per burst) and evidence of synchronized action potentials in the transmembrane potentials of the neuron population (Figure 3D–F).

In addition to our previous simulations, we also investigated a scenario where inhibitory control was absent. This can occur in situations such as selective apoptosis of interneurons or temporal disconnections between interneurons and pyramidal cells due to trauma [37]. In this absence of inhibition, the extracellular potentials reached even higher magnitudes (up to 14 mV) and the transmembrane potentials exhibited hyper-synchronization across the neural population (Figure 3G–I). This phenomenon can be attributed to the feedback loop between endogenous electric fields and neuronal activity, where a stronger electric field leads to increased synchronization, as demonstrated by the transition from partial synchronization (Figure 3F) to hyper-synchronization (Figure 3I) in the firing of action potentials. These findings support the notion that changes in inhibitory control can modulate electric field effects and subsequently influence the synchronization of neural activity.

Figure 4 presents the LFP series corresponding to different levels of inhibitory control as described earlier. As expected, at 100% inhibitory control (Figure 4A), no bursting patterns are observed in the LFP, with the voltage remaining below 0.35 mV. When inhibitory control is reduced to 50% (Figure 4B), a bursting pattern emerges, followed by a period of suppression. In the absence of inhibitory control (Figure 4C), the bursts increase in magnitude by roughly two times, and the duration between bursts is extended. These findings align with our experimental results (Figure 1), where the increase in burst magnitude corresponds to the decrease in inhibitory control, and we can attribute the longer suppression duration to the longer recovery time of extracellular calcium during seizures compared to lower magnitude bursts.

The emergence of burst suppression during the application of general anesthesia has long been studied; however, there is no general consensus on the mechanism. Starting with Kroeger and Amzica [38], it was believed that the burst suppression pattern reported in EEG occurred due to a phenomenon called “cortical hypersensitivity”, wherein neurons entered a hyper-excitable state once sufficient anesthetic was administered. It was shown that the period of suppression following a bursting event typically corresponds with the amount of time needed for extracellular calcium to recover to baseline levels. Along with calcium, Ching et al. [39], demonstrated through a dynamical model based on a network of Hodgkin– Huxley type neurons that neurometabolic dynamics can also play a role in the suppression phase of burst-suppression. Namely, during the burst event, ATP is rapidly consumed, causing action potential production to exceed consumption, which in turn opens ATP-gated potassium channels, triggering the suppression event. This suppression period terminates only once ATP concentrations return to the baseline level. The usefulness of the model lies in its applicability for interpreting burst suppression patterns beyond the application of anesthesia, as ATP dynamics can also be affected during scenarios of ischemic brain injury, hypothermia, and developmental encephalopathy, thus further expanding the etiology of burst suppression. Other notable models include the “mesoscopic” model of burst suppression, introduced by Liley et al. [40], which uses mean field theory to extend the fast dynamics occurring at the cellular level in order to model the dynamics at the populational, brain network level. By tuning certain parameters, they found that there exists a slow time scale of dynamics that modulates the fast activity of individual neurons, which can produce burst suppression-like activity. In contrast, our model encompasses both molecular and ephaptic mechanisms, providing a comprehensive explanation for all components of burst suppression. However, it is important to acknowledge that our model specifically considers the electric field effect arising from a decrease in the level of inhibition without factoring in other potential elements that could further influence the electric field. Any disruption in the proper functioning of the inhibitory system can lead to the generation of endogenous electric fields, which in turn can modulate neuronal firing rates and promote synchronization. This mechanism of electric field-mediated synchronization may underlie both burst suppression induced by anesthesia and epileptic activity. The differences in magnitude between these phenomena can be attributed to variations in the level of inhibition, which influences the strength of the electric field effect, in spite of the opposing effects on GABA receptors. Consequently, burst suppression and seizures, according to our results, share a common underlying mechanism with differences in their manifestation based on the degree of inhibition and the resulting electric field dynamics.

## Data Availability

The raw data and model code supporting the conclusions of this article will be made available by the authors upon reasonable request.

## Supplementary Materials

The following supporting information can be downloaded at: https://www.mdpi.com/article/10.3390/cells12182229/s1, Figure S1: Experimental setup; Figure S2: Time series of LFP band power, which correspond to Figure 2A; Figure S3: Neuronal activity with no electric field coupling; Figure S4: Tuning the stacking factor to match optogenetic stimulus data with computational model.
