# Supplementary material for: Electric Field Effects on Brain Activity: Implications for Epilepsy and Burst Suppression
Source: Cells. 2023 Sep 7;12(18):2229. doi: 10.3390/cells12182229 (PMC10527339; doi:10.3390/cells12182229)
Supplement: Supplementary file 1 [file cells-12-02229-s001.zip › cells-2492950-supplementary.pdf]

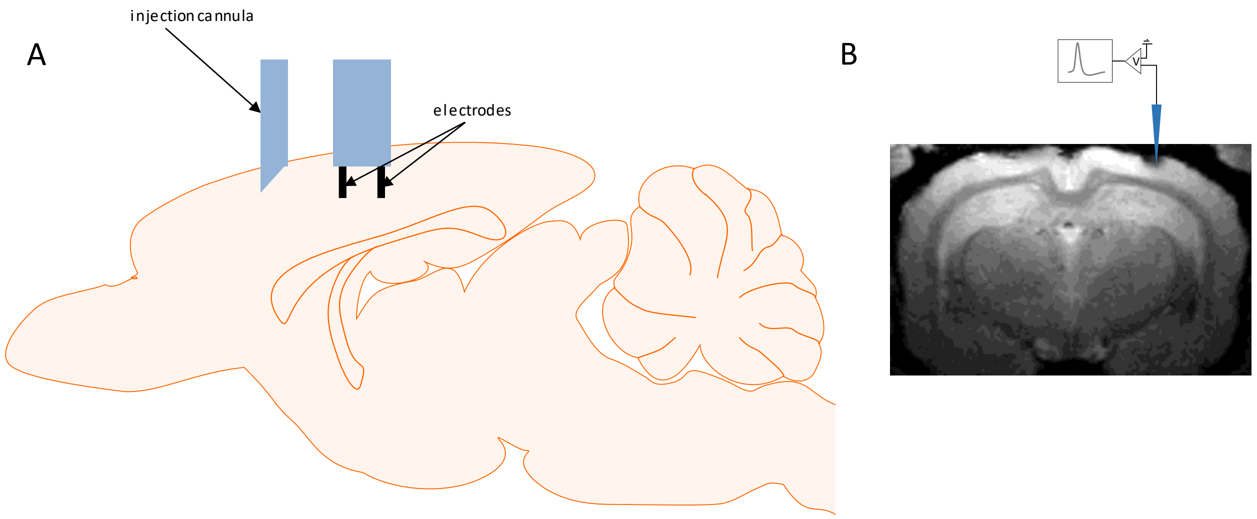

Figure S1. Experimental setup. The schematic illustrates the positioning of the implant (A) and the corresponding locations of the electrodes (B). Note that the area where the brain tissue is not visible is attributed to magnetic susceptibility artifacts.

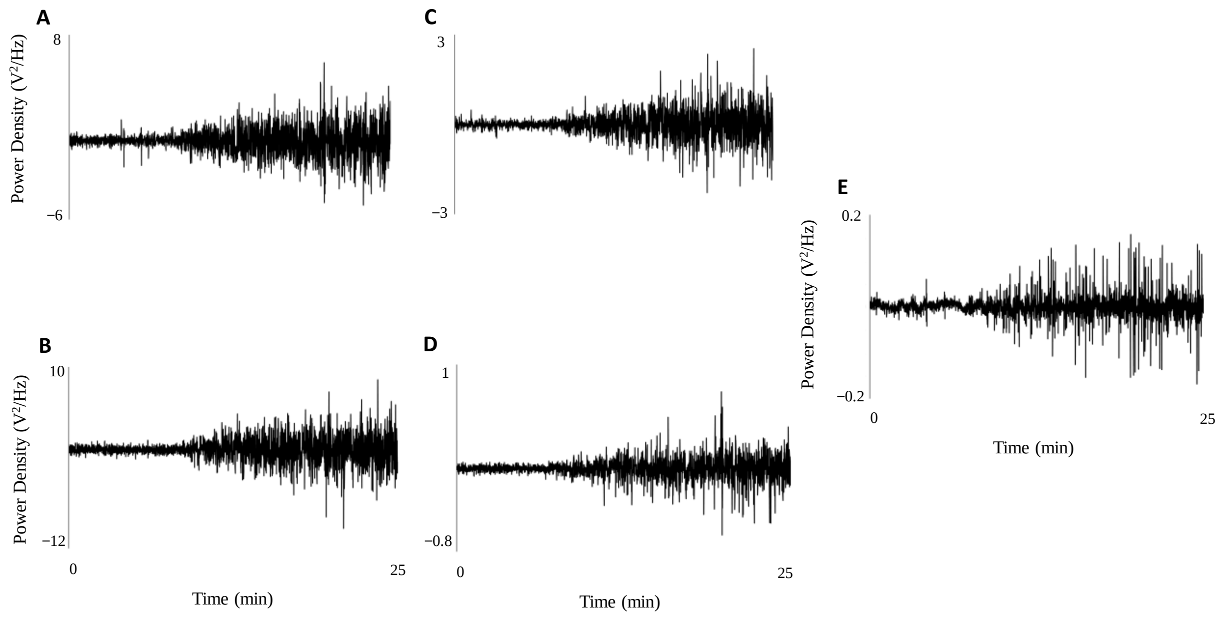

Figure S2: Time series of LFP band power, which correspond to Figure 2A. Using a rolling window power spectral density analysis, we show (A) delta band power, (B) theta band power, (C) alpha band power, (D) beta band power, and (E) gamma power.

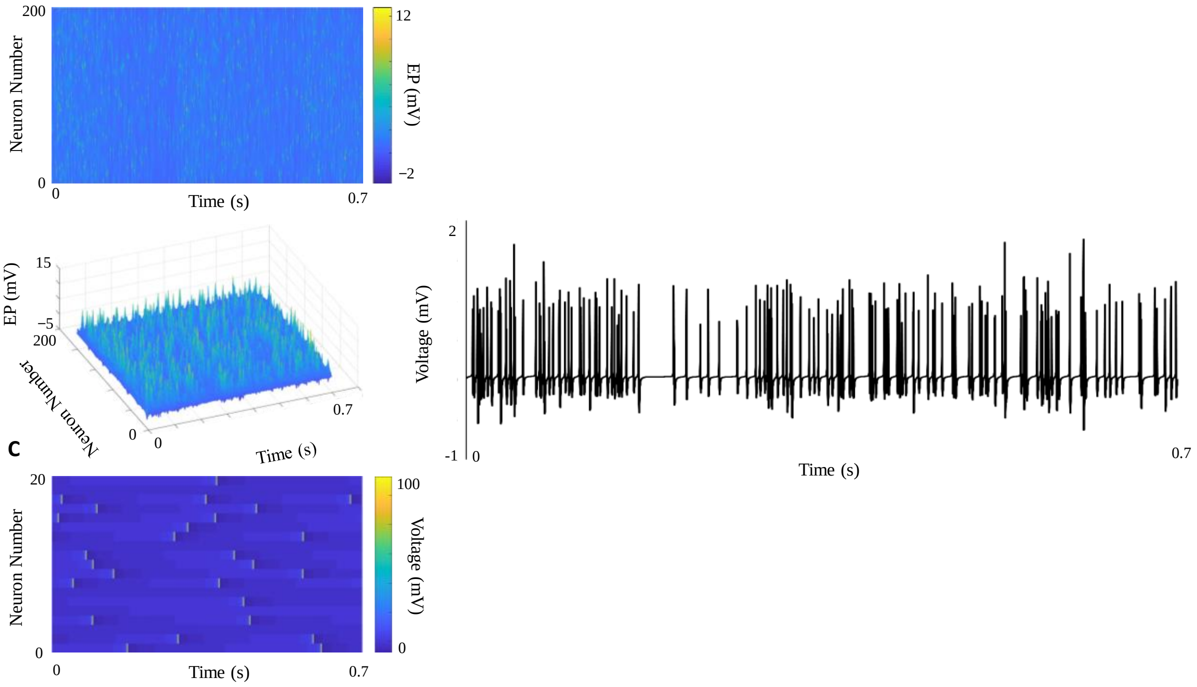

Figure S3: Neuronal activity with no electric field coupling. (A,B) Extra-cellular potential over 0.7 seconds with a stacking factor of 30 with no electric field coupling shows no synchronized activity. (C) Similarly, transmembrane potentials show no recruitment of cells despite the high stacking factor, since there is no ephaptic coupling. (D) Although the LFP magnitudes are higher than resting state, the lack of synchronization creates no bursts in the LFP recording.

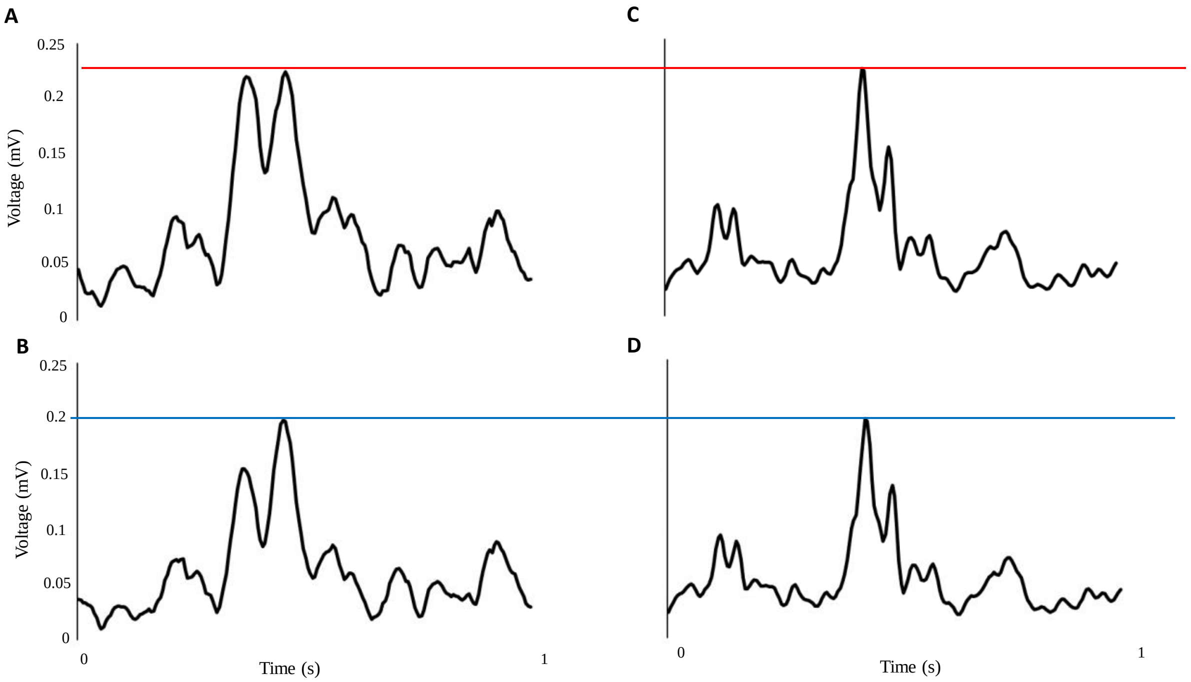

Figure S4: Tuning the stacking factor to match optogenetic stimulus data with computational model. Panels A, B show an example of a burst during optogenetic stimulation at (A) electrode 1 and (B) electrode 2. Since electrode 2 is further from the injected canula and the source of stimulated neuronal activity, the peak is somewhat attenuated. Panels C and D, which show the computational model with electrodes placed at (C) 100 microns and (D) 200 microns from the center of stimulation show that by selecting a stacking factor of 5.4, we are able to match the attenuation of the signal in respect to distance from the source. Red lines and blue lines show the peak value of the signal for electrode 1 and electrode 2.
